# An in-silico simulation study to generate computed tomography images from ultrasound data by using deep learning techniques

**DOI:** 10.1093/bjrai/ubaf005

**Published:** 2025-03-22

**Authors:** Anatol A Aicher, Davide Cester, Alexander Martin, Karolina Pawlus, Florian A Huber, Thomas Frauenfelder, Catherine Paverd

**Affiliations:** Institute for Diagnostic and Interventional Radiology, University Hospital Zurich, Rämistrasse 100, 8091 Zürich, Switzerland; Institute for Diagnostic and Interventional Radiology, University Hospital Zurich, Rämistrasse 100, 8091 Zürich, Switzerland; Institute for Diagnostic and Interventional Radiology, University Hospital Zurich, Rämistrasse 100, 8091 Zürich, Switzerland; Institute for Diagnostic and Interventional Radiology, University Hospital Zurich, Rämistrasse 100, 8091 Zürich, Switzerland; Institute for Diagnostic and Interventional Radiology, University Hospital Zurich, Rämistrasse 100, 8091 Zürich, Switzerland; Institute for Diagnostic and Interventional Radiology, University Hospital Zurich, Rämistrasse 100, 8091 Zürich, Switzerland; Institute for Diagnostic and Interventional Radiology, University Hospital Zurich, Rämistrasse 100, 8091 Zürich, Switzerland

**Keywords:** ultrasound, CT, cGAN, pix2pix, deep learning, augmentation, conversion, enhancement

## Abstract

**Objectives:**

Ultrasound has low sensitivity in parenchymal lesion detection compared to contrast-enhanced computed tomography (CT). In this proof-of-concept-study, we investigate whether raw ultrasound data can be used to generate CT-like images using deep learning models in order to enhance lesion detection.

**Methods:**

The k-wave ultrasound and Astra CT simulation toolkits were used to generate 2 datasets (1000 samples each) from simulated phantoms with up to 3 inclusions. The pix2pix conditional Generative adversarial network (cGAN) was trained on 800 samples per dataset, reserving the remainder for testing. Outputs were evaluated using generalized contrast-to-noise ratio (gCNR) and Structural Similarity Index (SSIM). Segmentation of B-mode alone versus B-mode with model-generated CT overlay was performed by 2 radiologists (1 board-certified and 1 resident) and both their performance and inter-observer agreement were evaluated using the Jaccard Index.

**Results:**

Model-generated CT-like images exhibited significantly improved gCNR (0.574±0.210 to 0.873±0.163) and SSIM (0.808±0.092 to 0.912±0.062) depending on phantom and inclusion type. Computed tomography-like images sometimes highlighted lesions otherwise undetectable in B-mode. The Jaccard index for 100 test samples improved significantly when segmenting Machine-Learning-augmented B-Mode compared with B-Mode images alone (0.58±0.18 to 0.69±0.16), depending on dataset and radiologist. Inter-observer agreement also improved significantly (0.74±0.18 to 0.85±0.07 for 1 dataset).

**Conclusions:**

Deep learning models can effectively translate ultrasound data into CT-like images, improving quality and inter-observer agreement, and enhancing lesion detectability, for example, by alleviating shadowing artefacts.

**Advances in knowledge:**

Generating CT-like images using raw ultrasound RF data as input to a cGAN model results in a significant improvement in lesion detectability by, for example, alleviating acoustic shadowing. With a cGAN architecture, even relatively small datasets can successfully generate CT-like images that improve lesion detectability.

## Introduction

Ultrasound is widely used as a first-line imaging technique as it is safe, non-invasive, and cost-effective. However, it faces limitations such as artefact susceptibility,^1^ inter-observer variability,^2^ operator dependency,^3^ and a trade-off between resolution and image depth,^4^ which limits its use in various diagnostic scenarios such as hepatocellular carcinoma detection,^5^ urolithiasis measurement,^6^ and imaging of obese patients.^7^ These challenges often necessitate alternative imaging techniques, notably computed tomography (CT) scanning, which offers detailed imagery but has drawbacks including the use of ionizing radiation and higher costs.

The ideal imaging modality would combine the advantages of ultrasound and CT. Advances in AI, particularly in image enhancement and translation, have the potential to improve ultrasound image quality. Generative adversarial networks (GANs),^8^ and subsequent developments like conditional GANs (cGANs)^9^ and the pix2pix model,^10^ have significantly advanced image generation. Pix2pix’s UNet architecture and PatchGAN discriminator lend themselves to medical imaging applications, evidenced by their use in SPECT denoising,^11^ fat suppression,^12^ MRI motion correction,^13^ and ultrasound beamforming.^14^ Moreover, traditional CNN-based image translation, such as the work by Vedula et al.,^15^ requires large datasets, limiting its applicability in medical contexts. In contrast, pix2pix has shown effectiveness with as little as a few hundred training images in some specific image translation tasks,^10^ suggesting that maybe useful results can be achieved with smaller datasets than traditionally assumed. However, this has not yet been extensively tested or verified in the context of clinical medical image translation.

Additionally, recent advances in computational power have enabled the accurate simulation of ultrasound and CT images based on custom phantoms. In the case of ultrasound, simulations enable access to raw radio frequency (RF) ultrasound datasets, prior to the signal processing required for generating traditional B-mode images, and allow for the application of machine learning (ML) algorithms to these RF signals. For example, several groups have used ML models on RF data to replicate computationally intensive image beamformers,^14^^,^^16–18^ or to train classifiers, for example, to distinguish glioma subtypes.^19^

Our paper presents a proof-of-concept work demonstrating the novel idea of translating raw ultrasound RF data to images resembling different imaging modalities in order to alleviate ultrasound limitations. We demonstrate an ultrasound-to-CT conversion technique utilizing the pix2pix model, trained and tested with simulated raw ultrasound RF data from the k-Wave^20^ toolbox, and simulated CT data from the ASTRA^21^ toolbox. We specifically use pix2pix as it is well-established, robust, fast, and has been shown to work in different image conversion tasks. We hypothesize that the application of this modern image translation network to ultrasound data will alleviate some ultrasound limitations, thus enhancing lesion visualisation and improving image quality.

## Methods

**Figure 1.**
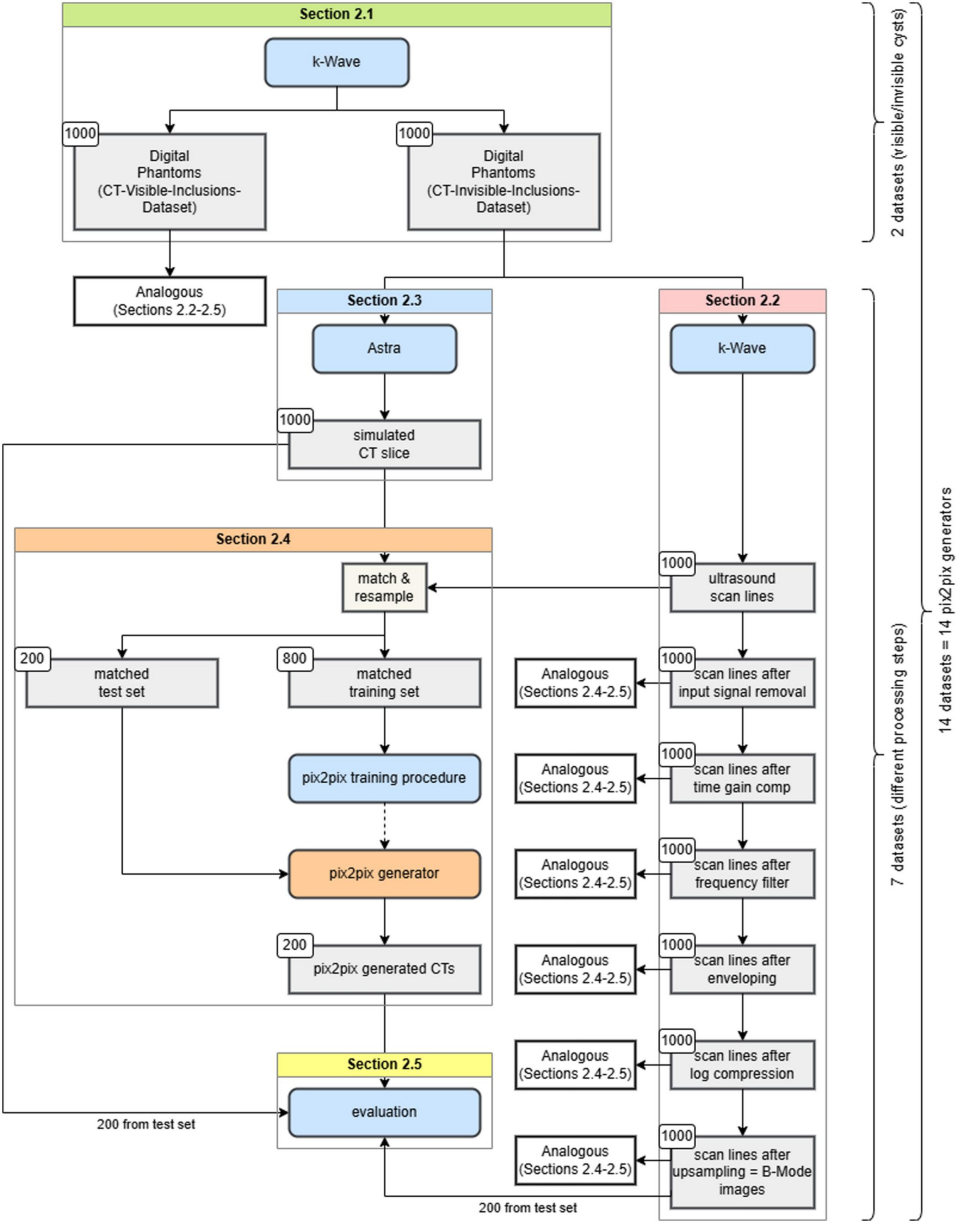
Flowchart depicting the methods used in this study, further discussed in detail in “Methods” subsections.

On Overview of the methods used can be seen in Figure 1.

### Phantoms

We generated 2 distinct phantom datasets using the k-Wave library (v1.3)^20^ for MATLAB (R2020b, the MathWorks Inc., MA USA). Each dataset contained 1000 phantoms, designed for both ultrasound and CT simulations. The 3D phantoms are cubes of background material with spherical inclusions, each pixel being assigned a random density and speed of sound, with mean and SD based on the material. The material properties of the phantoms can be found in Table 1, phantom and inclusion size as well as attenuation coefficient settings in Table 2, and the general characteristics are as follows:

**Table 1.** Physical properties of phantom materials.

	$c_{0}$ (m/s)	$\rho_{0}$ (kg/m^3^)	σ
Background CT-invisible-inclusions-dataset	1540	1000	0.008
Background CT-visible-inclusions-dataset	1520	1030	0.008
Inclusion (water)	1480	1000	0
Inclusion (muscle, along fibres)	1575	1065	0.08
Inclusion (kidney)	1570	1050	0.05

$c_{0}$ represents the mean sound speed, $\rho_{0}$ represents the mean density, and σ represents the relative SD within a material. Speed of sound and density values for inclusions are sourced from literature^39^ (Appendix A). The chosen relative SDs mimic ultrasound imaging behaviours in inclusions, ranging from anechoic (akin to medical imaging cysts) for water, to variably hyperechoic for muscle and kidney tissue inclusions.

**Table 2.** The properties used for phantom generation and ultrasound simulation with k-Wave.

Parameters for phantom generation and ultrasound simulation	
Parameter	Value
**Phantom**	
Phantom size (axial)	40 mm
Phantom size (transverse)	58.4 mm
Phantom size (elevation)	10 mm
Inclusion min. radius	1 mm
Inclusion max. radius	8 mm
Attenuation coefficient factor	0.75 db cm^−1^ MHz^−1^
Attenuation coefficient exponent	1.5
A/B	6
**Ultrasound simulation**	
Number of scan lines	128
Number of active transducer elements	32
Element size (transverse)	0.3 mm
Element size (elevation)	2.2 mm
Transducer source strength	2 MPa
Tone burst frequency	6 MHz
Focus distance	20 mm
Elevation focus distance	19 mm
Number of time steps	2934
Time step length (CT-invisible-inclusions-dataset)	57.1364 µs
Time step length (CT-visible-inclusions-dataset)	57.8882 µs

- CT-invisible-inclusions-dataset. Contains phantoms with some inclusions indistinguishable in CT images due to their mean density being identical to the background. The same inclusions, however, are visible on the B-Mode images, due to them having homogeneous material properties and thus being anechoic, akin to cysts in medical imaging.
- CT-visible-inclusions-dataset. Contains phantoms with all inclusions distinguishable in CT images, including the anechoic inclusions, which are set to have a density different to the background material.

The first dataset offered the possibility to see how the pix2pix algorithm deals with inclusions that are visible in one but not the other modality. The second dataset provided a comparison to a more ideal case, where every inclusion is, in principle, visible to both modalities.

### Ultrasound data

Simulated ultrasound images were generated using the GPU-accelerated k-Wave library from the parameters given in Table 2. The transmit apodization was set to a Hanning window, the receive apodization to a rectangular window. Scan lines produced from the k-Wave simulations were subsequently passed through the following processing stages: Input signal removal, time gain compensation, frequency filtering, envelope detection, log compression, and upsampling (yielding a B-Mode image). Following each stage of signal processing, new signal data matrices were generated from the channel data for input into the pix2pix model, yielding 7 distinct signal matrices of size 128 × 2934 (256 × 2934 after upsampling) per phantom.

### CT data

For CT data, we used the MATLAB and Python-based ASTRA Tomography Toolbox (v2.1).^21^^,^^22^ The phantoms, identical to those used in ultrasound simulations, were normalized and zero-padded for efficient GPU processing. Prior to input into the CT simulation code, effective windowing to density values between 0 and 1 was performed on the phantoms using the following pixel-based operation:

(1) $$P_{\mathrm{norm}}=\frac{\left(P_{\mathrm{orig}}-G_{\min}\right)}{\left(G_{\max}-G_{\min}\right)}$$

Using the ASTRA toolkit, we generated projection images from 100 scan angles. The Simultaneous Iterative Reconstruction Technique (SIRT) algortihm within the ASTRA toolkit was used for image reconstruction from the projection data, yielding 584 × 584 pixel CT images.

The simulated CT projection images showed both the expected artefacts and noise. An enlarged version of an exemplary simulated CT image showing these characteristics can be seen in the Supplementary Material.

### Image conversion

Image conversion was executed using the pix2pix model,^10^ implemented in Python (v3.8.10) with TensorFlow (v2.3.0). For a detailed architecture, refer to the Supplementary Materials Figures 1 and 2. The Adam optimizer was configured with parameters from the original pix2pix publication^10^: learning rate 0.0002, $\beta_{1}$ of 0.5, and $\beta_{2}$ of 0.999.

We aligned ultrasound data and CT images by cropping and then resampled them to 256 × 256 pixels to accommodate for pix2pix input restrictions, using the bilinear interpolation offered by the OpenCV python package (v4.2.0.34). Then both datasets were matched to create CT-ultrasound pairs across all processing stages. These pairs were divided into training (800 samples) and testing (200 samples) sets for the pix2pix model.

Pix2pix inputs underwent random jittering by up-sampling to 286 × 286 pixels, randomly cropping to 256 × 256 pixels, 50% probability of horizontal flipping, and normalization to [−1,1]. These per-sample processing steps were applied after assignment to the test/training sets, ensuring no mixing of datasets. The pix2pix model requires no feature selection.

The model was trained over 50 000 steps with a batch size of 1 and λ of 100 (as suggested in Phillip et al.^10^) on all 14 datasets separately, yielding 14 pix2pix models. Running these models on the 14 test sets (one for each processing step and each phantom type) yielded 14 sets of ultrasound-to-CT converted images. These were compared with their corresponding B-Mode and CT images for evaluation (see “Quality metrics” section).

### Quality metrics

To assess the quality of the generated images, we utilized the generalized contrast-to-noise ratio (gCNR) and the Structural Similarity Index (SSIM).^23^

The gCNR is defined as

(2) $$\text{gCNR}=1-\text{OVL}=1-\sum_{\text{bin}\in \text{bins}}\min(p_{\text{ROI},\text{bin}},p_{\text{BG},\text{bin}}),$$

where OVL denotes the overlap of the probability distribution p over the pixel intensity x for regions of interest (ROI) and background (BG). Generalized contrast-to-noise ratio provides a reliable quality measure unaffected by dynamic range adjustments and other post-processing adjustments.^24^ Generalized contrast-to-noise ratio was calculated separately for anechoic versus echogenic inclusions, considering the distinct signal quality of such inclusions in ultrasound images.

Structural Similarity Index, adapted from the Scikit-Image package (v0.17.2),^25^ was used to quantify the similarity between the generated and ground truth CT images.

### Clinical evaluation

In the final stage, a set of 200 images was examined by a board-certified radiologist as well as a resident with 4 years of experience in radiology. The image set contained matching pairs of B-Mode images with or without a pix2pix-generated CT overlay (refer to Figure 5), for 50 phantoms each from the CT-visible-inclusions-dataset and the CT-invisible-inclusions-dataset. The phantoms were selected randomly from all the phantoms containing at least one inclusion.

The order of the 200 images was randomized, and the radiologists were tasked with marking all inclusions on every image using freehand-drawn binary masks. The resulting masks were evaluated using the Jaccard index (intersetion-over-union),

(3) $$J=\frac{|A\cap B|}{|A\cup B|},$$

where A denotes one binary mask and B denotes another binary mask. The Jaccard index was selected because it is suitable for medical image segmentation tasks.^26^ We used it to compare the 200 binary masks of inclusions drawn by each of the 2 radiologists with the ground truth phantom inclusion masks. The resulting Jaccard indices were compared between images with versus images without the pix2pix-generated CT overlay.

### Statistical analysis

Statistical analysis was conducted to determine the significance of our quantitative findings. For Jaccard index, we used a dependent *t*-test for paired variables. For gCNR and SSIM datasets, requiring a multiple comparison test, preliminary tests using the Anderson-Darling and Levene tests revealed non-normal distribution and unequal variances. Therefore, we used the Kruskal-Wallis test followed by the Dunn test for our analyses, employing the Scipy package^27^ (v1.4.1) for Python for implementation.

## Results

**Figure 2.**
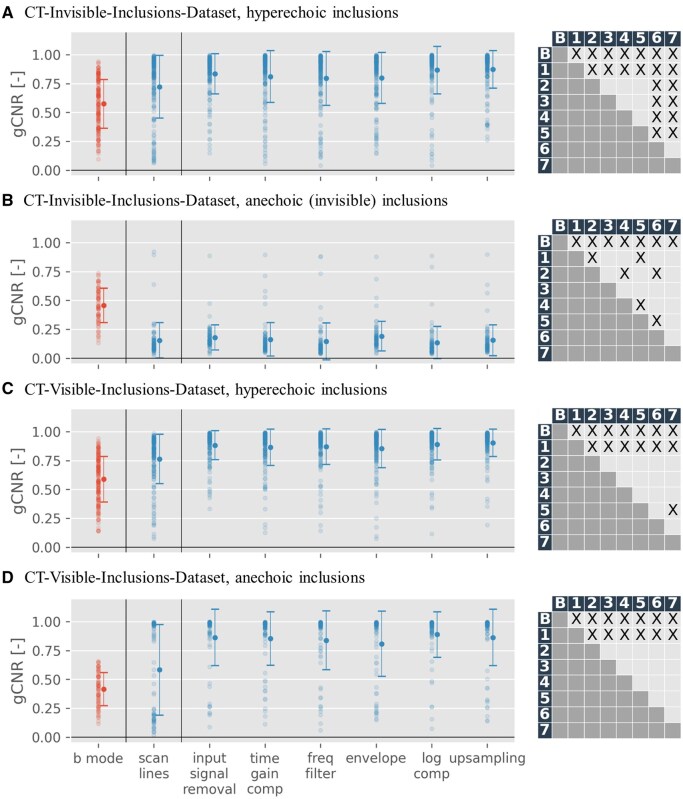
Generalized contrast-to-noise ratio (gCNR) scatter plots, depicting results for B-Mode images (red) against pix2pix outputs trained across all 7 input modalities (blue), for both datasets. The *x*-axis marks processing stages (“scan lines” correspond to raw data, “upsampling” to B-Mode images) of input images. On the right of each scatter plot is a grid depicting statistical significance for the difference of mean between pairs of gCNR-sets (label B referring to B-Mode images (red in the scatter plot) and labels 1-7 referring to outputs across all 7 input modalities (blue in the scatter plot)). X denotes statistically significant results (*P* < .05). A more detailed table showing mean and *P* values can be found in the supplementary material. For gCNR specifics, see “Quality metrics” section. Only phantoms with at least one inclusion are shown, in line with gCNR’s region-of-interest requirements, and results are categorized into anechoic and hyperechoic inclusions. Scan line results, omitted in Figure 5 for inferior performance, are separated by a line.

Figure 2 demonstrates improved gCNR with ultrasound-to-CT conversion, except for anechoic inclusions in the CT-invisible-inclusions-dataset, where gCNR is reduced.

Table 3 presents gCNR results quantitatively. Multiple comparison for statistical significance of differences of mean using Dunn’s test revealed that gCNR is significantly different for all generated CTs, regardless of input modality, when compared to B-Mode images (*P* < .001 for all except CT from scan lines in the CT-visible-inclusions-dataset for anechoic inclusions, where *P* < .05). There is also significant difference in gCNR between CTs generated from scan lines and CTs generated from all other input modalities, particularly for the CT-visible-inclusions-dataset and strong scatterers in the CT-invisible-inclusions-dataset (*P* < .05 for all). Finally, in the CT-invisible-inclusions-dataset, for strong scatterers, CTs from log compressed and from upsampled input data are significantly different from all other input modalities, but not from each other (*P* < .05 for all). Full results of multiple comparison analyses can be found in supplements.

**Table 3.** gCNR values for simulated B-Mode image and pix2pix-generated CT images.

gCNR								
	B-mode	Scan lines	Input signal removal	Time gain compensation	Frequency filter	Envelope	Log compression	Upsampling
**CT-invisible-inclusions-dataset**								
Hyperechoic inclusions	0.57 ± 0.21	0.72 ± 0.27	0.83 ± 0.17	0.81 ± 0.23	0.80 ± 0.23	0.80 ± 0.23	0.87 ± 0.20	0.87 ± 0.16
Anechoic inclusions	0.46 ± 0.15	0.15 ± 0.15	0.18 ± 0.11	0.16 ± 0.14	0.14 ± 0.16	0.19 ± 0.13	0.14 ± 0.14	0.15 ± 0.13
**CT-visible-inclusions-dataset**								
Hyperechoic inclusions	0.59 ± 0.20	0.76 ± 0.21	0.88 ± 0.13	0.86 ± 0.16	0.87 ± 0.15	0.85 ± 0.17	0.89 ± 0.14	0.90 ± 0.12
Anechoic inclusions	0.41 ± 0.14	0.58 ± 0.39	0.86 ± 0.39	0.85 ± 0.23	0.84 ± 0.26	0.81 ± 0.28	0.89 ± 0.20	0.86 ± 0.24

Mean ± SD of generalized contrast-to-noise ratio values for the simulated B-Mode image (leftmost column, grey) as well as pix2pix-generated computed tomography images. The rows denote either anechoic or hyperechoic inclusions as regions of interest, for either the CT-invisible-inclusions-dataset or the CT-visible-inclusions-dataset. Columns 2-8 denote generated CTs using each of the 7 processing steps as input data.

**Figure 3.**
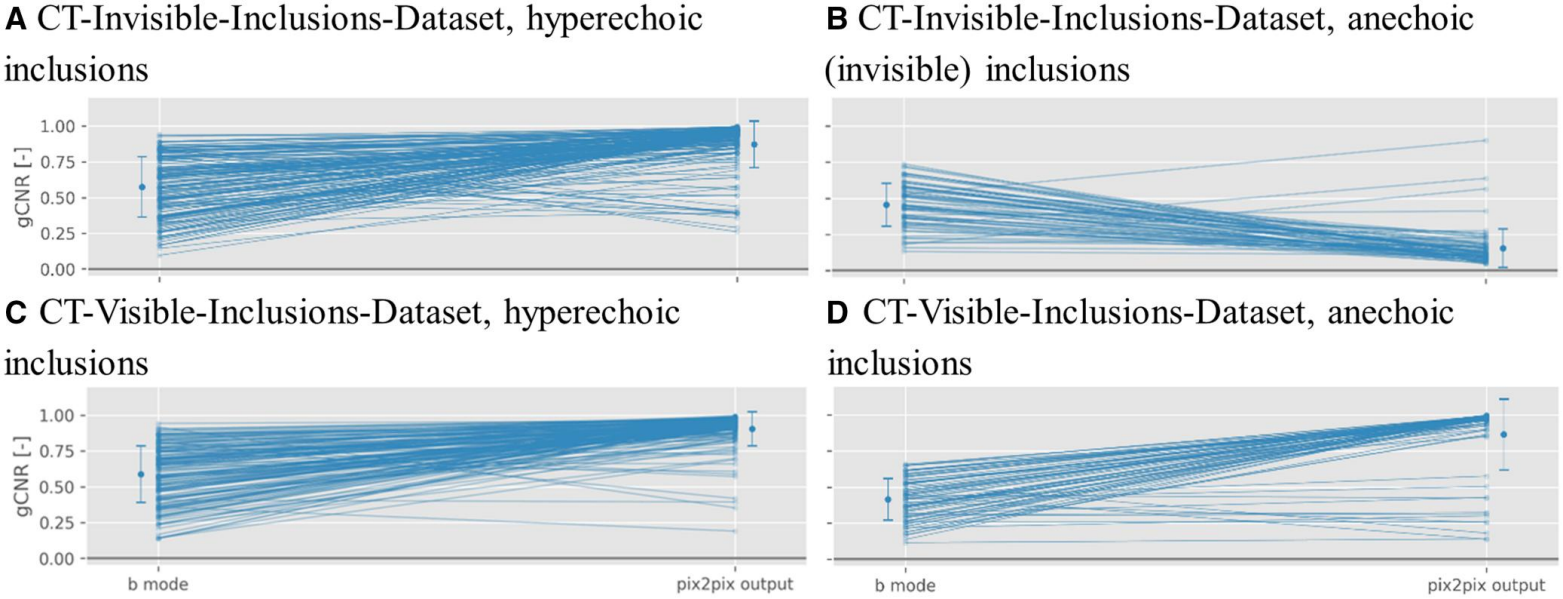
This scatter plot illustrates the generalized contrast-to-noise ratio (gCNR) change for phantoms with more than zero inclusions. The left column presents the gCNR of the input B-Mode image, while the right details the gCNR of the model-generated computed tomography images using the B-Mode as input. These data points mirror the first (red) and last columns in Figure 2.

Figure 3 reveals a gCNR increase in almost all phantoms when compared to the B-Mode image, with limited exceptions (and an inverse effect seen with anechoic inclusions in the CT-invisible-inclusions-dataset).

**Figure 4.**
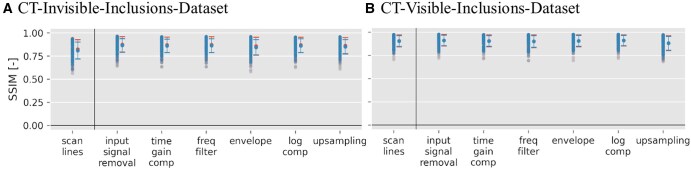
Structural Similarity Index (SSIM) scatter plots for pix2pix outputs when trained on both datasets across all 7 input signal data matrices. Blue symbols represent phantoms with 1 or more inclusions, and red symbols signify phantoms with 0 inclusions. For more details on the SSIM metric, refer to “Quality metrics” section. Scan line results are separated by a line because they were not used in Figure 5 due to their inferior performance in the generalized contrast-to-noise ratio metric (see Figure 2)

For the SSIM quality metric, Figure 4 showcases that it remains consistently high across different input stages regardless of the number of inclusions.

Numerical results of SSIM for B-Mode to CT conversion are available in Table 4, showing that values are between 0.808±0.092 and 0.912±0.062, for the invisible- and CT-visible-inclusions-dataset, respectively.

**Table 4.** SSIM values for the pix2pix-generated CT image.

SSIM							
	Scan lines	Input signal removal	Time gain compensation	Frequency filter	Envelope	Log compression	Upsampling
**CT-invisible-inclusions-dataset**							
0 inclusions	0.91 ± 0.06	0.91 ± 0.06	0.91 ± 0.06	0.90 ± 0.07	0.91 ± 0.06	0.91 ± 0.06	0.88 ± 0.08
>0 inclusions	0.90 ± 0.06	0.91 ± 0.06	0.90 ± 0.06	0.90 ± 0.06	0.91 ± 0.06	0.91 ± 0.06	0.88 ± 0.07
**CT-visible-inclusions-dataset**							
0 inclusions	0.82 ± 0.10	0.88 ± 0.08	0.87 ± 0.08	0.87 ± 0.08	0.86 ± 0.09	0.87 ± 0.08	0.86 ± 0.09
>0 inclusions	0.81 ± 0.09	0.86 ± 0.07	0.86 ± 0.07	0.86 ± 0.07	0.84 ± 0.08	0.86 ± 0.07	0.85 ± 0.08

Mean ± SD of Structural Similarity Index (SSIM) values for the pix2pix-generated CT using different processing steps for inputs. The rows denote phantoms that either contain 0 inclusions, or more than 0 inclusions, for either the computed tomography (CT)-invisible-inclusions-dataset or the CT-visible-inclusions-dataset. Columns denote generated CT images using each of the 7 processing steps as input data.

**Figure 5.**
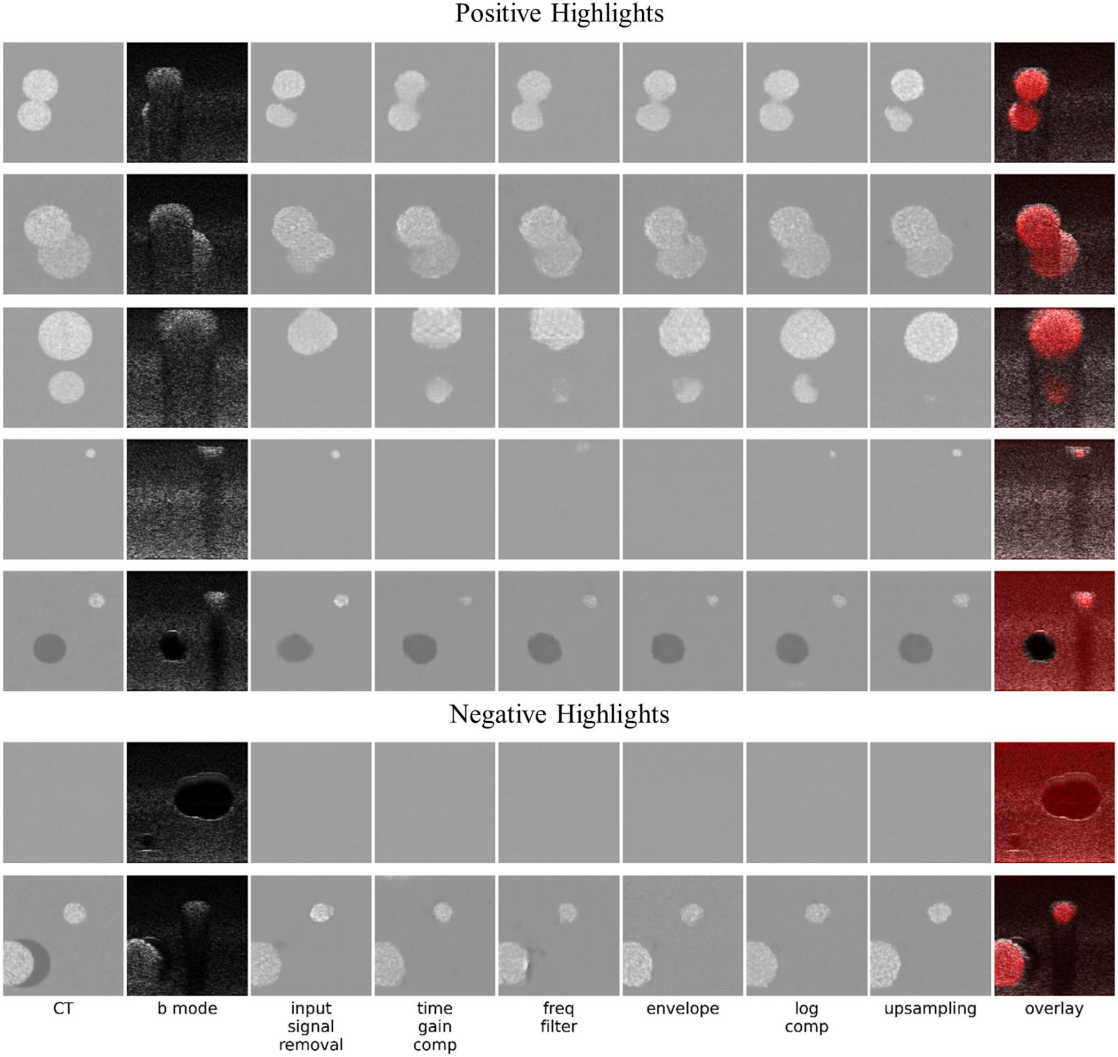
This figure shows some positive (rows 1-5) and negative (rows 6 and 7) highlights of image conversion, chosen as exemplary for various qualitative observations made. Column 1 (left) shows ground truth simulated computed tomography (CT) images. Column 2 shows simulated B-mode images. Columns 3-8 show the output of the ML model when trained on different stages of the ultrasound signal processing pipeline: after input signal removal, time gain compensation, frequency filtering, envelope detection, log compression, and upsampling (results using raw scan lines as input are omitted due to their inferior performance, see Figure 2). The final column shows generated CT images in red (using the average image intensities of the 6 previous columns, normalized to cover the entire dynamic range) superimposed on a B-Mode image.

For a qualitative assessment, we provide select phantoms’ transformation outputs, underlining both the successes and constraints of the ultrasound-to-CT conversion, found in Figure 5.

Rows 1-3 underscore the enhancement of visibility for inclusions. Markedly, row 3 highlights the detection of previously undetectable inclusions. Row 4 offers a refined spatial resolution perspective close to the transducer (where the focus is weak, leading to spread-out inclusions on the ultrasound), while row 5 showcases successful differentiation between anechoic and echogenic inclusions.

Figure 5 also demonstrates cases of less ideal image conversion. In row 6, using the CT-invisible-inclusions-dataset, pix2pix mimics CT’s limitations, obscuring anechoic inclusions otherwise visible in B-Mode. In row 7, from the CT-visible-inclusions-dataset, pix2pix overlooks an anechoic inclusion near another inclusion. Overall, it can also be noted that the generated CTs tend to not quite precisely capture the shapes of the inclusions.

**Figure 6.**
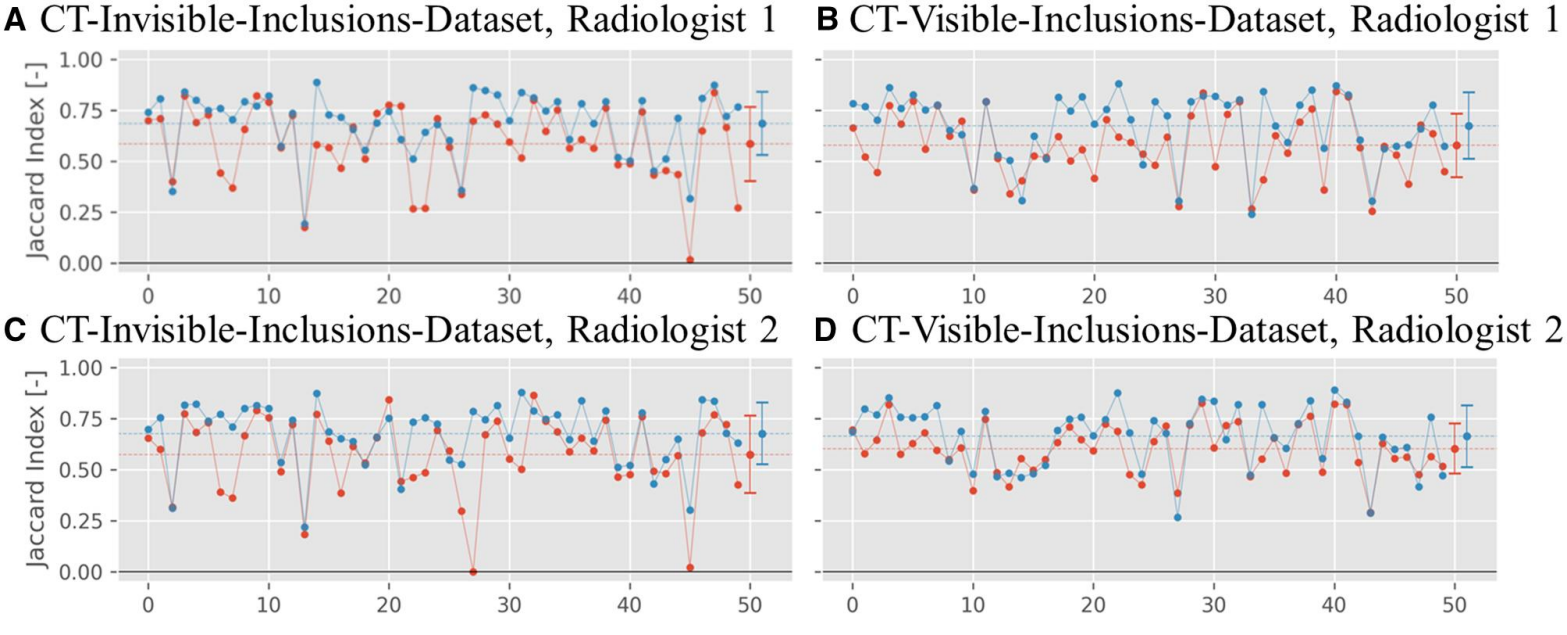
Jaccard index plots for the binary inclusion maps created by the 2 radiologists, when comparing to the ground truth. Red symbols indicate Jaccard indices for B-Mode images, and blue symbols indicate Jaccard indices for ML-augmented B-Mode images (as in Figure 5). The Jaccard indices are paired to be from the same phantom. Lines between points do not indicate interpolation but are rather meant to aid in comparing pairs of values. The horizontal dashed line indicates the mean of each set of Jaccard indices.

Figure 6 shows values for the Jaccard index for the two datasets segmented by 2 radiologists. For the CT-invisible-inclusions-dataset, the mean increases from 0.58±0.18 for B-Mode only to 0.69±0.16 for ML-augmented B-Mode for radiologist 1, and increases from 0.57±0.19 to 0.68±0.15 for radiologist 2. Similarly, for the CT-visible-inclusions-dataset, the mean increases from 0.58±0.16 to 0.67±0.16 for radiologist 1 and from 0.60±0.12 to 0.66±0.15 for radiologist 2. All differences are statistically significant with *P* < .05.

**Figure 7.**
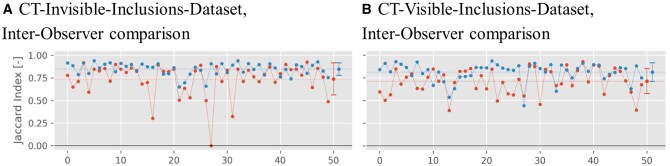
Jaccard index plots for the binary inclusion maps created by the 2 radiologists, when comparing with each other. Red symbols indicate Jaccard indices for B-Mode images, and blue symbols indicate Jaccard indices for ML-augmented B-Mode images (as in Figure 5). The Jaccard indices are paired to be from the same phantom. Lines between points do not indicate interpolation but are rather meant to aid in comparing pairs of values. The horizontal dashed line indicates the mean of each set of Jaccard indices.

Figure 7 shows the values for the Jaccard index comparing the segmentations between both radiologists for either dataset. For the CT-invisible-inclusions-dataset, the mean increases from 0.74±0.18 for B-Mode only to 0.85±0.07 for ML-augmented B-Mode. Similarly, for the CT-visible-inclusions-dataset, the mean increases from 0.71±0.14 to 0.81±0.10. All differences are statistically significant with *P* < .001.

## Discussion

Our results highlight the utility of the pix2pix model in translating ultrasound images to CT-like visualizations using signal data from different stages of the ultrasound image processing pipeline.

Quantitatively, improved gCNR and consistently high SSIM values demonstrate that pix2pix model-generated CTs align well with the corresponding ground truth CT images, while also enhancing specific image quality metrics compared to B-Mode images. Qualitatively, model-generated CTs exhibit a close resemblance to their ground truth counterparts. More importantly, our results demonstrate the ability to visualize some inclusions that were previously undetectable in ultrasound images. For example, the model performed well in reducing artefacts such as acoustic shadowing, which enabled the accurate visualization of lesion borders (Figure 5, row 2), or of entire lesions (Figure 5, rows 1 and 3) that were previously obscured. The ability of pix2pix to remove shadows has previously been demonstrated,^28^ and it is interesting to note that this strength of pix2pix remains in the context of “acoustic shadowing.” This removal of acoustic shadowing in particular holds significant clinical potential in various contexts. In liver imaging, for example, the presence of acoustic shadows from ribs has led to difficult-to-learn work-around techniques, such as using mirror artefacts for lesion detection.^29^ In breast cancer imaging, retroareolar shadowing is still a major problem in lesion detection,^30^ hindering accurate ultrasound-based mammography screening. Further examples can be found in thyroid cancer assessment, where shadowing precludes the assessment of the margins of calcified nodules,^31^ or in the sizing of kidney stones,^32^ which is a critical factor in urolithiasis management, or in the assessment of gallbladder contents in porcelain gallbladder, where calcification results in partial or complete shadowing.^33^ Any technique to help remove these limitations from acoustic shadowing could improve the sensitivity and specificity of ultrasound assessment in these, and other, clinical contexts.

The model also showed variable lesion identification performance at different signal-processing stages. For example, for small lesions close to the transducer, models trained on signal data after the input signal removal stage, or from the log compression stage onwards, have the best performance (Figure 5, row 4). This may be due to the high relative amplitude of signals in the region close to the transducer prior to time gain compensation, or the more even distribution of signal amplitudes from weak scatterers following log compression.

Overall, the positive results in lesion detection suggest that the model can offer a notable improvement in ultrasound imaging. This is further supported by assessment by 2 clinicians. The Jaccard index, which was calculated based on segmentations performed by either of the 2 radiologists, was statistically significantly higher for ML-augmented B-Mode images versus standard B-Mode images (Figure 6). In addition, the Jaccard index comparing segmentations from both radiologists to each other was also higher for the ML-augmented B-Mode images, showing better inter-observer agreement (Figure 7). This demonstrates that the ML-augmented images allow for more accurate segmentations across different skill levels for both an attending and a resident radiologist. Further, it enables the resident to achieve segmentations more similar to the ones performed by an attending (board-certified) radiologist.

Nevertheless, in some phantoms the model performance is inadequate. Some lesions that were visible on original ultrasound images were not discernible on model-generated CTs. This signifies a fundamental challenge in translating from ultrasound to CT; not all structures that are visible in ultrasound are visible in CT. Therefore, direct translations between modalities may obscure certain lesions. Instead, combining the model-generated CT output with the B-Mode image, as illustrated in our overlays (Figure 5), offers a more comprehensive view of the tissue and pathological structures. Importantly, we did not see any lesions in model-generated CTs that were not present in the ground truth.

Additionally, not all lesions that were invisible on B-Mode could be revealed by image translation. For example, the model did not perform well when identifying very small lesions hidden by acoustic shadowing and did not always accurately capture lesion shapes. These results may be improved by modifying the original pix2pix algorithm to accept significantly larger, non-square matrices, which would remove the need to downsample the ultrasound data (thereby retaining all spectral content). Furthermore, pix2pix is a general model with application in many fields; however, designing a specific model that exploits the signal structure of RF data (eg, when designing loss functions) could further improve performance.^34^

One methodological takeaway from this research is that even using a small dataset, we were able to improve image quality and increase the ability of radiologists to segment inclusions accurately. Thus, it is possible that in very specific cases a limited amount of training data, combined with the correct ML model, could still produce useful results. Although this is promising, especially in medical imaging where obtaining large datasets can be difficult, significant further work is required to evaluate the amount of data required to train models such as pix2pix using real clinical data.

Reproducibility in machine learning remains an active research area, with many existing and evolving standards.^35^ Kapoor and Narayanan^36^ elaborate on data leakage as one source of irreplicability. We have avoided the 3 types of loss they discuss, by: first, cleanly separating the test and training dataset; second, using only ultrasound data as input features; and third, avoiding any inter-dependence between phantoms or sampling bias. Other authors highlight concerns over both reproducibility and data accessibility.^37^^,^^38^ Due to the size of the datasets, our data are available directly on request from the authors. A relevant limitation of our study concerning reproducibility is the lack of a standardized and portable environment.

Additional study limitations relate to complexity and choice of ML-model. First, it is an in-silico study utilizing phantoms with a maximum of 3 round inclusions centered in the elevational plane, and limited possibilities for density and speed of sound. In reality, human tissue is much more complex, with a larger range of physical properties, and with tissue transitions at arbitrary angles and shapes. Second, pix2pix was designed as a general model, and therefore may demonstrate limited performance compared to models specifically designed for raw ultrasound data.

Overall, while this study outlines a promising approach to improving ultrasound imaging, significant work is still required before it can be translated into actual clinical applications. This includes testing and refining this new approach in simulation studies with more realistic targets as well as on real data acquired from physical phantoms and human patients. Additionally, any clinical application of our technique in future should be tested with a larger number of radiologists and clinicians from different specialties across different skill levels and from different institutions.

This study demonstrates the process of converting ultrasound data to CT-like images with a simple general purpose cGAN. The goal is to encourage further research into the novel concept of using raw ultrasound data as input into image-conversion deep learning models. Future work will investigate ultrasound-to-CT image conversion using specialized ML models and real-world data. Further development will be completed in a portable environment for seamless reproducibility and evaluated by a larger and more diverse set of clinicians.

## Supplementary Material

ubaf005_Supplementary_Data
