# Supplementary material for: An in-silico simulation study to generate computed tomography images from ultrasound data by using deep learning techniques
Source: BJR Artif Intell. 2025 Mar 22;2(1):ubaf005. doi: 10.1093/bjrai/ubaf005 (PMC13045719; doi:10.1093/bjrai/ubaf005)
Supplement: ubaf005_Supplementary_Data [file ubaf005_supplementary_data.pdf]

Supplementary Material

Model architecture

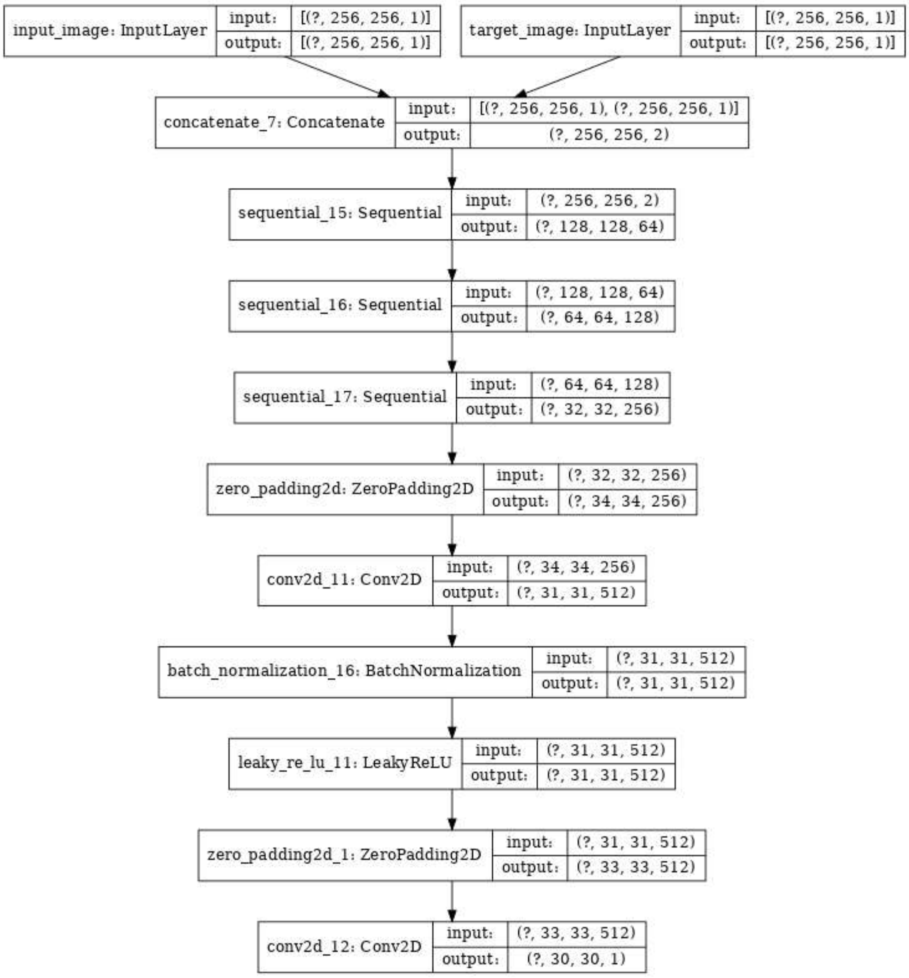

Supplemental Figure 1. The architecture of the discriminator used in our pix2pix implementation. The discriminator consists of a PatchGAN. The question marks denote variable batch sizes. In the original pix2pix publication (10), a batch size of 1 was determined to be ideal and thus used in this work.

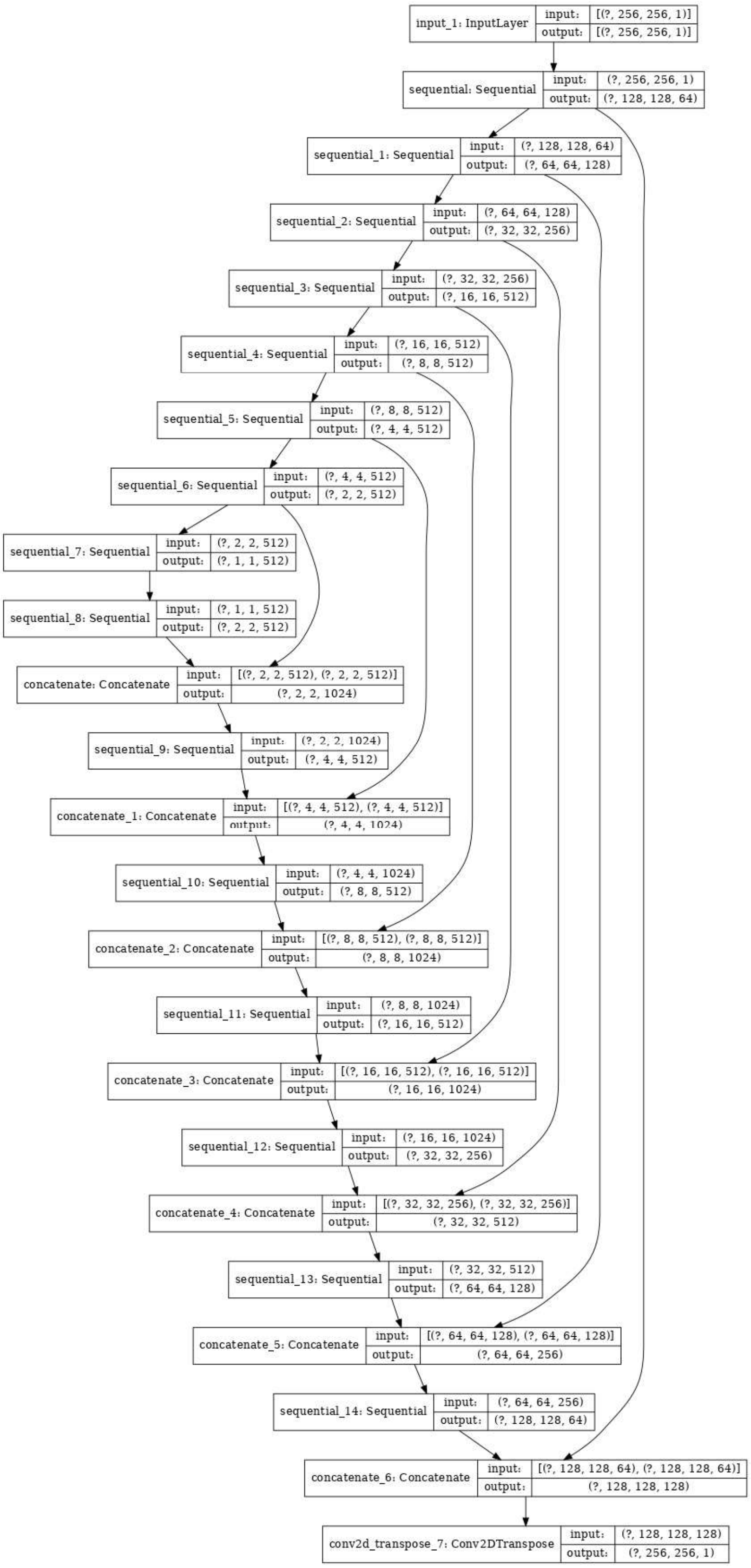

Supplemental Figure 2. The architecture of the generator used in our pix2pix implementation. The generator consists of an encode-decode network with skip connections between layer i and $n-i$, i.e. a Unet. The question marks denote variable batch sizes. In the original pix2pix publication (10), a batch size of 1 was determined to be ideal and thus used in this work.

A CT-Invisible-Inclusions-Dataset, hyperechoic inclusions

	b mode	scan lines	input signal removal	time gain comp	freq filter	envelope	log comp	upsampling
b mode	0.57 ± 0.21	2.55e-09	9.55e-22	1.80e-23	1.92e-19	8.05e-21	4.76e-42	9.73e-38
scan lines	2.55e-09	0.72 ± 0.27	6.55e-03	1.73e-03	3.10e-02	1.23e-02	3.02e-12	5.63e-10
input signal removal	9.55e-22	6.55e-03	0.83 ± 0.17	1.00	1.00	1.00	1.50e-03	1.45e-02
time gain comp	1.80e-23	1.73e-03	1.00	0.81 ± 0.23	1.00	1.00	5.87e-03	4.20e-02
freq filter	1.92e-19	3.10e-02	1.00	1.00	0.80 ± 0.23	1.00	1.46e-04	2.78e-03
envelope	8.05e-21	1.23e-02	1.00	1.00	1.00	0.80 ± 0.22	6.25e-04	7.97e-03
log comp	4.76e-42	3.02e-12	1.50e-03	5.87e-03	1.46e-04	6.25e-04	0.87 ± 0.20	1.00
upsampling	9.73e-38	5.63e-10	1.45e-02	4.20e-02	2.78e-03	7.97e-03	1.00	0.87 ± 0.16

B CT-Invisible-Inclusions-Dataset, anechoic (invisible) inclusions

	b mode	scan lines	input signal removal	time gain comp	freq filter	envelope	log comp	upsampling
b mode	0.46 ± 0.15	1.92e-21	6.16e-10	1.25e-18	3.18e-27	1.20e-09	1.83e-28	5.94e-20
scan lines	1.92e-21	0.15 ± 0.15	2.36e-02	1.00	1.00	1.73e-02	9.02e-01	1.00
input signal removal	6.16e-10	2.36e-02	0.18 ± 0.11	1.50e-01	1.60e-04	1.00	5.26e-05	6.65e-02
time gain comp	1.25e-18	1.00	1.50e-01	0.16 ± 0.14	5.17e-01	1.20e-01	3.05e-01	1.00
freq filter	3.18e-27	1.00	1.60e-04	5.17e-01	0.14 ± 0.16	1.03e-04	1.00	8.44e-01
envelope	1.20e-09	1.73e-02	1.00	1.20e-01	1.03e-04	0.19 ± 0.13	3.28e-05	5.10e-02
log comp	1.83e-28	9.02e-01	5.26e-05	3.05e-01	1.00	3.28e-05	0.14 ± 0.14	5.48e-01
upsampling	5.94e-20	1.00	6.65e-02	1.00	8.44e-01	5.10e-02	5.48e-01	0.15 ± 0.13

C CT-Visible-Inclusions-Dataset, hyperechoic inclusions

	b mode	scan lines	input signal removal	time gain comp	freq filter	envelope	log comp	upsampling
b mode	0.59 ± 0.20	1.64e-09	3.68e-37	1.82e-34	3.24e-36	1.18e-29	2.24e-45	8.67e-49
scan lines	1.64e-09	0.76 ± 0.21	1.64e-09	3.21e-08	4.52e-09	5.75e-06	7.12e-14	8.85e-16
input signal removal	3.68e-37	1.64e-09	0.88 ± 0.13	1.00	1.00	1.00	1.00	6.10e-01
time gain comp	1.82e-34	3.21e-08	1.00	0.86 ± 0.16	1.00	1.00	6.17e-01	2.10e-01
freq filter	3.24e-36	4.52e-09	1.00	1.00	0.87 ± 0.15	1.00	1.00	4.44e-01
envelope	1.18e-29	5.75e-06	1.00	1.00	1.00	0.85 ± 0.17	7.47e-02	1.34e-02
log comp	2.24e-45	7.12e-14	1.00	6.17e-01	1.00	7.47e-02	0.89 ± 0.14	1.00
upsampling	8.67e-49	8.85e-16	6.10e-01	2.10e-01	4.44e-01	1.34e-02	1.00	0.90 ± 0.12

D CT-Visible-Inclusions-Dataset, anechoic inclusions

	b mode	scan lines	input signal removal	time gain comp	freq filter	envelope	log comp	upsampling
b mode	0.41 ± 0.14	7.26e-03	7.39e-17	1.37e-12	5.07e-13	5.93e-12	4.24e-15	5.74e-16
scan lines	7.26e-03	0.58 ± 0.39	3.95e-06	1.10e-03	6.54e-04	2.38e-03	4.28e-05	1.34e-05
input signal removal	7.39e-17	3.95e-06	0.86 ± 0.24	1.00	1.00	1.00	1.00	1.00
time gain comp	1.37e-12	1.10e-03	1.00	0.85 ± 0.23	1.00	1.00	1.00	1.00
freq filter	5.07e-13	6.54e-04	1.00	1.00	0.84 ± 0.26	1.00	1.00	1.00
envelope	5.93e-12	2.38e-03	1.00	1.00	1.00	0.81 ± 0.28	1.00	1.00
log comp	4.24e-15	4.28e-05	1.00	1.00	1.00	1.00	0.89 ± 0.20	1.00
upsampling	5.74e-16	1.34e-05	1.00	1.00	1.00	1.00	1.00	0.86 ± 0.24

Supplemental Figure 3. Results for gCNR, for the CT-invisible-inclusions-dataset. The diagonal (grey background) shows mean gCNR ± standard deviation. The off-diagonal elements show the p-value comparing respective gCNR values with a Dunn-Test, after a Kruskal-Wallis test showed significance to $p < 0.001$ for all datasets. Green background shows values below 0.001, orange shows values between 0.001 and 0.05. Red shows values above 0.05. "b mode" refers to the simulated ultrasound b mode image, the other rows and columns refer to pix2pix-generated CTs using the respective processing step as an input.

Additional Results

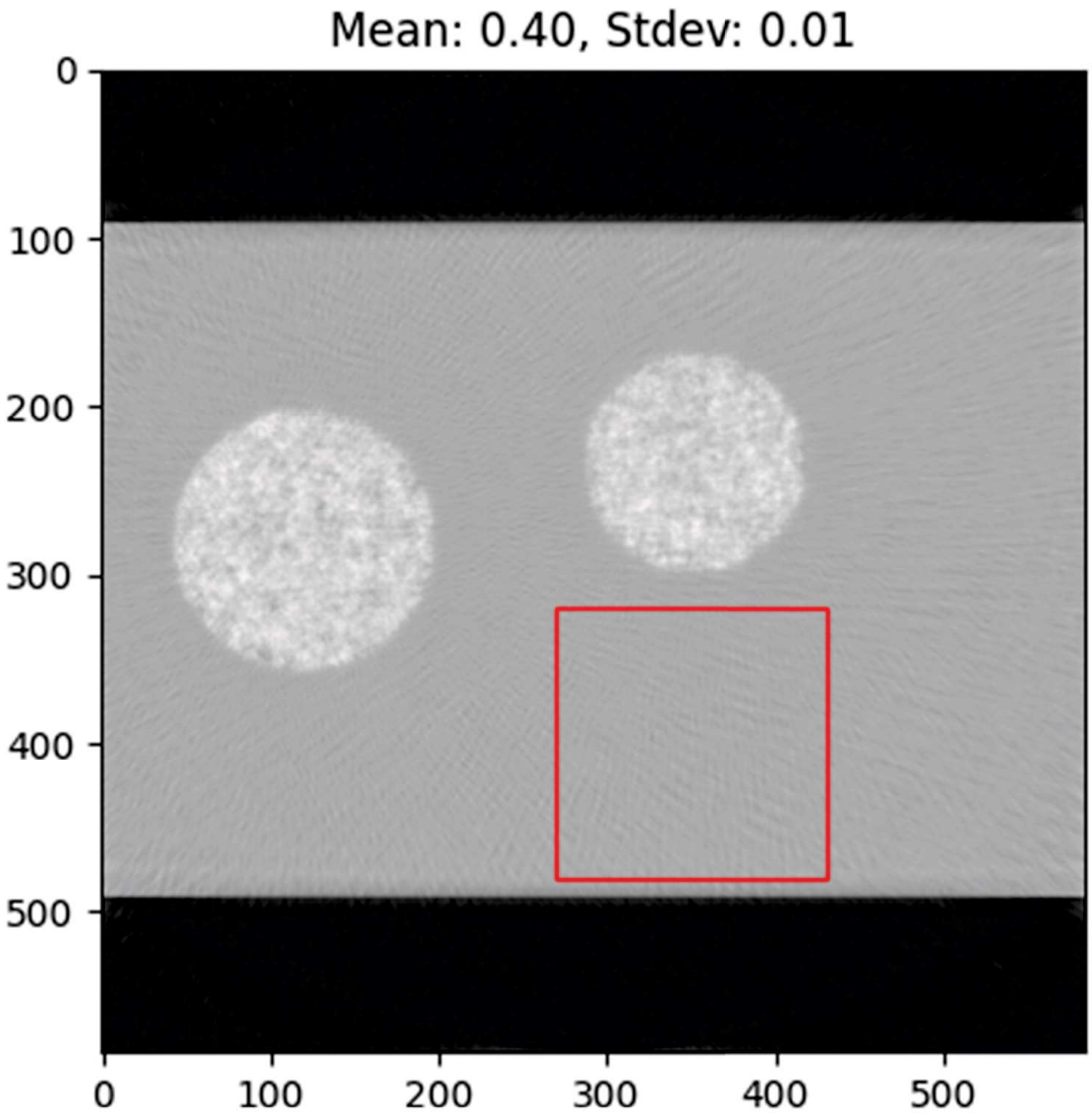

Supplemental Figure 4. An example ct image as generated with ASTRA toolkit. The mean and standard deviation noted in the image are for the red square, which was chosen to only contain background material.

CT data
